# Meat-inspection in Small Cities and Towns1Read before the meeting of the Pennsylvania State Veterinary Medical Association, Scranton, September, 1899.

**Published:** 1899-11

**Authors:** John J. Repp

**Affiliations:** Veterinarian, State Experiment Station, Ames, Iowa


#THE JOURNAL

OF

### COMPARATIVE MEDICINE AND VETERINARY ARCHIVES.

VOL. XX. *NOVEMBER, 1899.* No. 11.

### MEAT-INSPECTION IN SMALL CITIES AND TOWNS.[1]

BY JOHN J. REPP, V.M.D.,

VETERINARIAN, STATE EXPERIMENT STATION, AMES, IOWA.

WE hear a great cry nowadays for meat-inspection in large cities. I have not one word of objection. Nothing but my best wishes for success and earnest endeavor for its attainment are offered to those who are promoting the establishment of such inspection; but if the large cities, why not the small cities and boroughs?

Almost every reason for inspection of food animals in large cities can be urged for inspection in cities and towns of smaller size. Certainly, the health and lives of the people are as important in the one place as in the other. So far as desires go, I feel certain that as large a percentage of the population in small cities and towns are acquainted with the danger of consuming uninspected meat, and are as anxious to have it inspected as in the large cities.

So far as the needs of inspection are concerned, I am certain that the needs of the one are as great as those of the other. In fact, in small places the butchers are more apt to purchase and kill animals that are unfit for consumption than is the case in large cities. The tendency is for farmers and stock owners to hold the best animals, such as fat steers and heifers, choice lambs and hogs, for sale in the city markets, while they offer old bulls and cows and sheep and swine that do not fatten well to the local butcher for slaughter. It is, of course, true that many local butchers are content only with the best class of food animals. However, almost every small city and town has its unprincipled butcher, who makes a specialty of slaughtering and selling for food animals that are of inferior quality and often unfit for human consumption. I am personally

[1] Read before the meeting of the Pennsylvania State Veterinary Medical Association, Scranton, September, 1899.

acquainted with enough such cases to fully warrant the statements I have made. I believe that the unprincipled butcher in the country town has a better opportunity to perpetrate his nefarious crimes upon an unwary public than has his city brother of the same persuasion, and I doubt not that he uses it.

Furthermore, many an honest and conscientious butcher puts upon his block meat unfit for food because he is ignorant of the fact that it is unfit. He kills a fat animal that has tuberculosis or trichinosis without the knowledge that it is so affected. If he does notice a lesion he passes it by as of no importance. Because we understand these diseases and their danger to health and life we should not hastily infer that the butcher is acquainted with them. Also, a butcher may notice a diseased state of the animal before slaughter, or a lesion in the carcass, but because he does not want to lose it by voluntary condemnation, he consoles himself with the probably conscientious thought that it is of no great importance, and allows it to go into his refrigerator, and thence into the stomachs of his customers. In these ways, and in others, meat that ought to be condemned is consumed, and thus disease is spread and perpetuated.

Agreeing, then, that we need inspection, how shall we get it? It is necessary for us to educate the people so that they may have knowledge of their danger. This done, meat-inspection will abound everywhere. When the people see the need of a thing and ask for it, it must come. In this land *vox populi, vox Dei.* How can we educate the people? It is not difficult. The veterinarian in the rural town has an easier task than his co-worker in the city. Let him speak to the people privately and at public meetings; let him write articles for the town papers. Everybody in town reads the papers, so this affords an easy means of access to the people. If this be done, and the people be taught what they should demand, only the plan of giving them what they want yet remains to be devised. If there is co-operation extensive enough the matter may be controlled by State legislation. The State laws in many cases require that only pure food shall be put upon the market, even that a certain fertilizer sack must contain the ingredients of such kind and percentage as stated on the sack. Then why not require that meat, which should be only pure and wholesome, shall possess these qualities. Failing State legislation, the city or borough ordinance may be invoked. The local veterinarian should be the meat inspector. His salary should be fixed according to his duties and paid by public taxation of the people by whom he is employed. He should be required to be present on killing days at the various places used for slaughter. He should examine the animal both before and after death, and should condemn at one time or the other all animals not fit for food. The killing days can easily be so fixed in towns of the size under consideration that the inspector can be present when killing is done. Let it be required that a tag be placed on all meat that has passed inspection. A penalty should be imposed upon the butcher for evasion of the law or ordinance.

The matter of assumption of the loss of condemned carcasses offers a more serious problem for solution. The provision might be made that if the butcher purchases an animal in which disease or unfitness for slaughter is obvious he shall bear the loss. Also, that if the animal looks before slaughter as if it might be a fit subject for food, but on slaughter turns out not to be so, let the State, or county, or city, or borough under whose law or ordinance the system is operated pay the cost of the animal to the butcher. It may be said that this will entail a heavy loss on the taxpayers. It may be answered that if we want the assurance of good, wholesome meat we must be willing to pay for the execution of a plan that will give it to us. Money spent by the people in this way would be well spent, and I do not believe they will complain.

In the town where I live, with a population of 2500, there are two butchers. They each use twenty to twenty-five animals a week. They kill on Monday and Friday, as a rule. Under such circumstances the local practising veterinarian could easily arrange his work so that he could be present at times when animals are slaughtered. If he were paid, say, five hundred dollars per year he would, perhaps, be well paid for his services. This would be easily borne by the taxpayers, and in return they would have the assurance of good and wholesome meat. I have no doubt they would feel that they are getting the full value for the cost entailed.

In case of larger towns of say 5000 to 15,000 population, the killing could all be provided for in one central abattoir, where all the improved facilities could be used. In this way one inspector could readily do all the inspecting. The same arguments for this arrangement for slaughter hold in such places as in our large cities.

It may be argued that much of the meat consumed in small cities and towns is obtained at the great slaughter-houses in the cities, and if these are not under inspection, people living in the former places will be under the necessity of using uninspected meat. It may be answered that it looks now as if our large cities will have a system of inspection before our small ones. In this event the argument falls to the ground. Even if we should secure a system of inspection before the large cities, other things equal, conditions would be much as now. At the present time meat from the large slaughter-houses is so high in price that local butchers cannot handle it, as they can supply home-dressed beef at a much lower price. Even before the recent decided advance in beef a dealer in my town, who sold only city-dressed beef, was forced out of business entirely, because he could not compete in price with local butchers and sell his meats at a profit. Furthermore, if no inspection existed in the cities and there was inspection in rural places, the city uninspected meats could not find sale in competition with the inspected product of rural cities and towns, because the people would demand meats of the latter class. For this reason, if for no other, we would have nothing to fear from the uninspected meats of large cities.

With this I leave the subject with you for discussion. If this hastily prepared paper will set some of us to more serious thinking and acting, it will have done all that I can hope for it.

